# Molecular Genomic Study of Inhibin Molecule Production through Granulosa Cell Gene Expression in Inhibin-Deficient Mice

**DOI:** 10.3390/molecules27175595

**Published:** 2022-08-30

**Authors:** Hira Sajjad Talpur, Zia ur Rehman, Mostafa Gouda, Aixing Liang, Iqra Bano, Mir Sajjad Hussain, FarmanUllah FarmanUllah, Liguo Yang

**Affiliations:** 1 National Center for International Research on Animal Genetics, Breeding and Reproduction (NCIRAGBR), Huazhong Agricultural University, Ministry of Science and Technology of the People’s Republic of China, Wuhan 430070, China; 2 Department of Animal Breeding and Genetics, Sindh Agriculture University, Tandojam 70060, Sindh, Pakistan; 3 College of Veterinary Sciences, Faculty of Animal Husbandry and Veterinary Sciences, University of Agriculture, Peshawar 25120, Khyber Pakhtunkhwa, Pakistan; 4 College of Biosystems Engineering and Food Science, Zhejiang University, Hangzhou 310058, China; 5 Department of Nutrition & Food Science, National Research Centre, Dokki, Giza 12622, Egypt

**Keywords:** FSH, DEGs, granulosa cells, hCG, inhibin, Inha

## Abstract

Inhibin is a molecule that belongs to peptide hormones and is excreted through pituitary gonadotropins stimulation action on the granulosa cells of the ovaries. However, the differential regulation of inhibin and follicle-stimulating hormone (FSH) on granulosa cell tumor growth in mice inhibin-deficient females is not yet well understood. The objective of this study was to evaluate the role of inhibin and FSH on the granulosa cells of ovarian follicles at the premature antral stage. This study stimulated immature wild-type (WT) and Inhibin-α knockout (Inha−/−) female mice with human chorionic gonadotropin (hCG) and examined hCG-induced gene expression changes in granulosa cells. Also, screening of differentially expressed genes (DEGs) was performed in the two groups under study. In addition, related modules to external traits and key gene drivers were determined through Weighted Gene Co-Expression Network Analysis (WGCNA) algorithm. The results identified a number of 1074 and 931 DEGs and 343 overlapping DEGs (ODEGs) were shared in the two groups. Some 341 ODEGs had high relevance and consistent expression direction, with a significant correlation coefficient (r^2^ = 0.9145). Additionally, the gene co-expression network of selected 153 genes showed 122 nodes enriched to 21 GO biological processes (BP) and reproduction and 3 genes related to genomic pathways. By using principal component analysis (PCA), the 14 genes in the regulatory network were fixed and the cumulative proportion of fitted top three principal components was 94.64%. In conclusion, this study revealed the novelty of using ODEGs for investigating the inhibin and FSH hormone pathways that might open the way toward gene therapy for granulosa cell tumors. Also, these genes could be used as biomarkers for tracking the changes in inhibin and FSH hormone from the changes in the nutrition pattern.

## 1. Introduction

Gonadotropins are glycoprotein hormones produced in the pituitary by gonadotropic cells the lightest copious ovarian cells and regulate ovarian follicle development [1,2]. These peptide hormones are also known for regulating ovarian and testicular function and are essential for normal growth, sexual development, and reproduction. Also, gonadotropins include some essential hormones like inhibin, FSH, and luteinizing hormone (LH) [2,3]. For instance, inhibin is released from the ovarian granulosa cell. It is a heterodimeric glycoprotein that consists of α-subunit linked with a β-hormone [4]. It is a member of the transforming growth factor-β superfamily that stimulate the release of FSH from the pituitary cells. Moreover, inhibin plays an important role in the FSH feedback secretion regulation during puberty in females [5]. In which, its secretion changes during female puberty have disclose correlation with adulthood chronic diseases like diabetes type 2 and heart disease which are generally considered among the common malnutrition diseases [6]. In addition, the inhibin hormone is essential as a diagnostic marker for ovarian cancer [7,8]. For instance, Rathore, et al. [9] mentioned that Inhibin deficiency in mice leads to the growth of gonadal sex cord-stromal tumors. In such cases, the mice mostly died at the age of 28 days due to testicular and ovarian tumors with cachexia-related signs [10,11,12]. A genetic method assured that gonadotropins play a key modifier role for gonadal sex cord-stromal tumor growth in inhibin-deficient mice. In that study, Nagaraja, et al. [13] reported that inhibin genetically interacts with multiple factors that influence testicular and ovarian growth and differentiation, including the pituitary gonadotropins [14]. Furthermore, they found a complex interplay among inhibins, gonadotropins, and ovarian cancer. Thus, the tracking of inhibin and FSH hormones could enhance the diagnostic performance of the health-related disease, not just ovarian female disease.

Furthermore, it is known that granulosa cells (GCs) are somatic cells of the sex rope that is mainly related to an embryonic female gamete identified to be an oocyte that exists in the animals’ ovary [15]. GCs show various phenotypes in the follicle, reliant on their position. Additionally, GCs gene expression analysis is very important to understand its functional mechanism that is related to animal growth. Hence gene association networks are very essential for expressing the relationship patterns between genes transversely microarray data, where the weighted gene co-expression network analysis (WGCNA) algorithm is a necessary tool to determine the relationship patterns among the genes microarray data [16], for which, the WGCNA algorithm is frequently used to understand the genes’ molecular processes and identification of interrelated genes and modules [17]. Therefore, it can show the co-expression structure and cluster the expression data into modules of conserved function that allow one to detect patterns of gene connectivity that can be aligned with behavioral and physiological phenotypes [18]. Meanwhile, PCA is a multivariate statistical procedure that uses an orthogonal transformation to convert a set of observations of possibly correlated variables into a set of values of linearly uncorrelated variables called principal components [19,20,21].

Therefore, this study aimed to investigate the variations in hCG-induced gene expression in WT and Inha−/− granulosa cells. It provided a novel reference for the pathway mechanisms. Initially, differentially expressed genes (DEGs) in WT/Inha−/−with and without human chorionic gonadotropin (hCG) stimulation samples were identified. Then the overlapping genes as the characteristic genes in hCG stimulation and Inha−/− female mice granulosa cells were selected and further investigated. For this process, the WGCNA algorithm was used to identify the interrelated gene patterns. Besides, PCA was used to examine the important regulated genes. The examined outcome recommended that Inhibin α knockout and hCG stimulation can down-regulate JUP expression, and up-regulate Psmc3ip expression. These genes could be used as a marker for the early prediction of granulosa cell carcinomas.

## 2. Results

### 2.1. Hierarchical Clustering and Comparison Analysis of Selected DEGs in Different Groups

**Figure 1:**
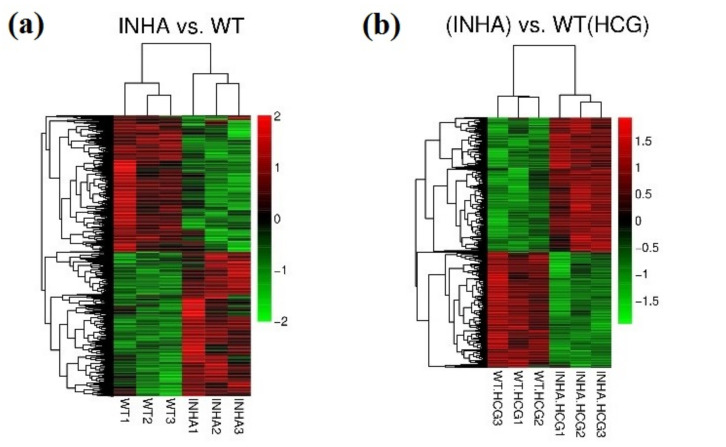
Hierarchical clustering heatmap of DEGs in group Inha−/− vs. WT (**a**) and Inha−/− (hCG) vs. WT(hCG) (**b**).

We extracted DEG expressions from two groups and drew hierarchical clustering heatmaps, as shown in Figure 1. The experimental samples in each group were divided into two separate parts, indicating that DEGs had obviously different expression patterns in each group (gene expression matrix can be found in Table S1, Supplementary Materials).

### 2.2. GO and KEGG Pathway Enrichment Analysis for the ODEGs

**Figure 2:**
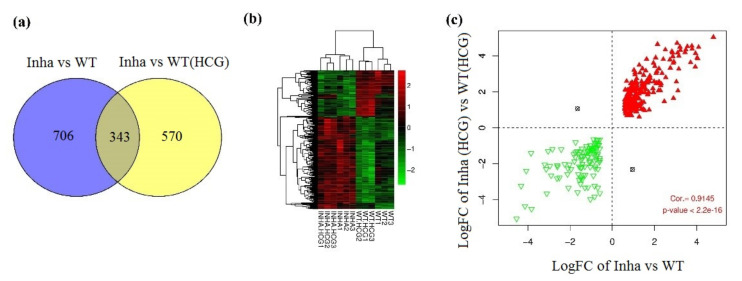
(**a**) Venn diagram of DEGs in groups Inha−/− vs. WT and Inha−/− (hCG) vs. WT(hCG). (**b**) Hierarchical clustering heatmap of overlapping DEGs. (**c**) Scatter-plot of correlation between logFC of Inha−/− vs. WT and Inha−/− (hCG) vs. WT(hCG). The red triangle and green inverted triangle refer to up and down-regulated DEGs in both Inha−/− vs. WT and Inha−/− (hCG) vs. WT(hCG).

**Figure 3:**
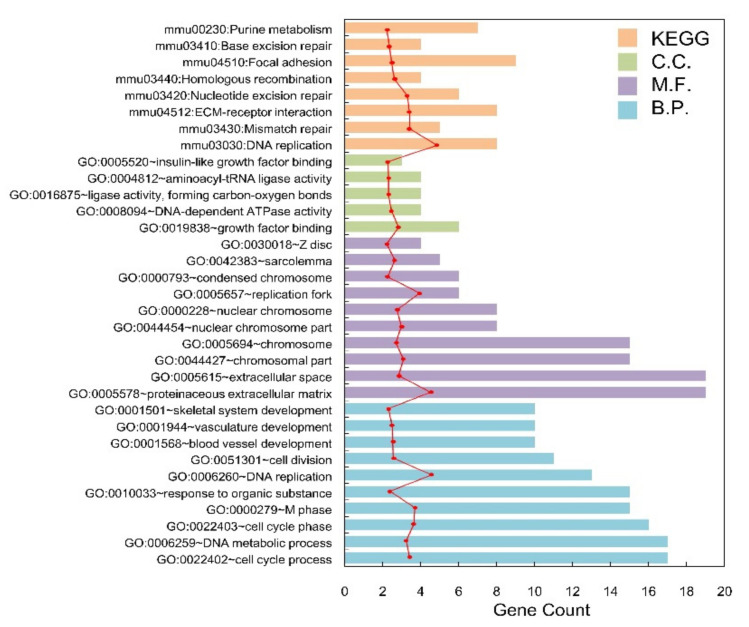
The histogram of the category of enriched GO terms and KEGG pathways for the overlapped DEGs. The horizontal axis represents the number of blue, purple, green, and orange mean Biology Process, Cellular Component, Molecular Function, and pathways, respectively; the red dot curve means –log10 (*p*-value).

Figure 2a shows the Venn diagram of DEGs in groups Inha−/− vs. WT and Inha−/− (hCG) vs. WT(hCG). In which, 343 overlapped genes were found with a total of 25 (10 BP, 10 CC, 5 MF) significant related GO annotations for 343 overlapped genes that listed in Table 1. Also, the hierarchical clustering heatmap of ODEGs showed a significant negative correlation of the down-regulated Inha DEGs compared to the WT group (Figure 2b,c). What’s more, among the 343 shared DEGs, 341 had a consistent expression direction and high relevance, with a significant correlation coefficient of 0.9145 (*p* < 0.000001). The shared 343 DEGs were differentially expressed in both Inha−/− vs. WT group and Inha−/− (hCG) vs. WT(hCG) group (Table S1). Meanwhile, the GO BPs were significantly related to the cell cycle process (GO: 0022402), which had the most enrichment significance (*p*-value < 0.0001). Also, 17 genes were involved, such as CDC6, KIFC1, MKI67, DSN1, NUF2. Besides, according to KEGG enrichment analysis, ODEGs were significantly enriched in eight pathways (RFC5, PRIM1, RPA2, RFC4, LIG1, POLD2, POLE, MCM2) participating in the most significant related pathway: DNA replication (mmu03030). Using the ggplot2 package in R, significant related GO and KEGG pathways annotations were displayed in Figure 3.

### 2.3. Physiological Phenotypes R Modules and Genes Identification Based on WGCNA

**Figure 4:**
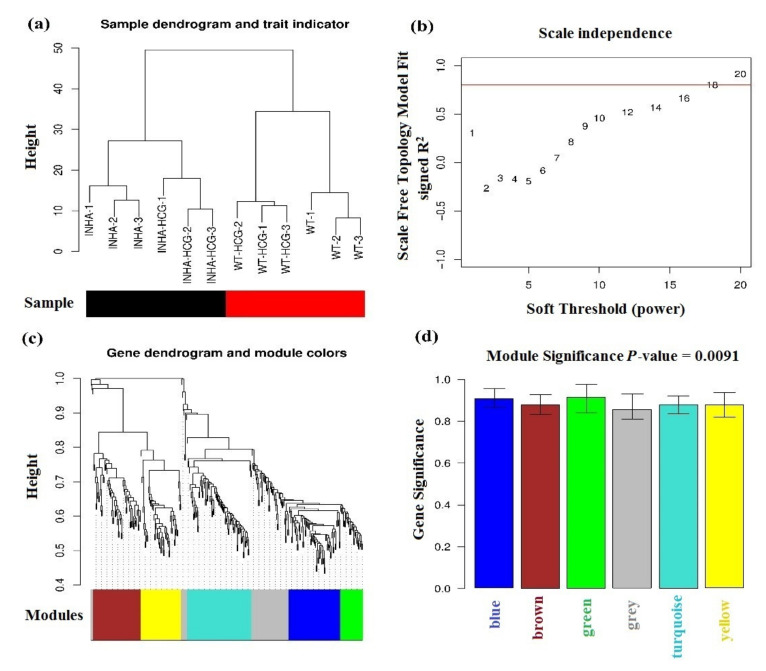
(**a**) Sample clustering tree. The Black and red bars mean different types of samples. (**b**) power-law coefficient parameter plot X-axis means soft-threshold, Y-axis means scale-free topology model fit signed R-square. (**c**) Modules clustering tree, different colors in the bottom mean different modules. (**d**) Module bar plot, X-axis means different modules, Y-axis means the significance of genes in each color module based on their different physiological phenotypes.

For clustering the ODEGs based on the physiological phenotypes, the expression data were processed by square root transformation and used to infer co-expression gene network modules with the WGCNA network construction and the module detection method. Firstly, the distances among all the samples were studied to eliminate discrete samples with no discrete samples to be removed (Figure 4a). In which, a high affinity between Inha and Inha(hCG) was found by the clustering tree. Then a proper power-law coefficient was selected using the soft-threshold method (Figure 4b). Through this model the selected soft-threshold (X-axis) was 18 when the scale-free topology model fit was signed at correlation coefficient (R^2^) = 0.8 (Y-axis). Then, a dynamic hierarchical tree cut algorithm was used to detect the co-expression modules, and a total of six related modules were found (Figure 4c). Moreover, the R^2^ between the physiological phenotypes and each module had a very high correlation coefficient (over 0.8) with the physiological phenotypes (Figure 4d and Table 2). Thus, 153 genes in the top three modules (blue, green, and brown) in total were selected as representative ODEGs for further analysis based on their significant physiological phenotypes.

### 2.4. Co-Expression Network Construction

**Figure 5:**
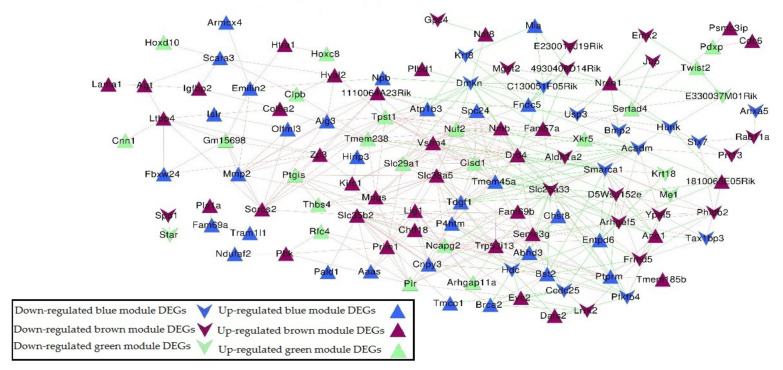
Gene co-expression network based on 153 DEGs in blue, green, and brown modules. Triangle and inverted triangle refer to up and down-regulated DEGs; blue, green, and brown nodes mean genes from the corresponding colored module. Redline connections mean a positive correlation coefficient, and green line connections mean a negative correlation coefficient.

The Co-expression Network had 153 DEGs in blue, green, and brown modules, as well as an expression correlation coefficient from the WGCNA algorithm (expression correlation matrix was shown in Table S2). Also, the selected gene pair was based on the expression correlation coefficient >0.8 as shown in Figure 5. The co-expression network included 122 nodes in total. These nodes had 35 down-regulated genes (14 blue, 17 brown, and 4 green) and 87 up-regulated genes (30 blue, 36 brown, and 21 green genes) with 410 edges (129 negative coefficient connections and 281 positive coefficient connections) (Table 2 and Table S3).

**Figure 6:**
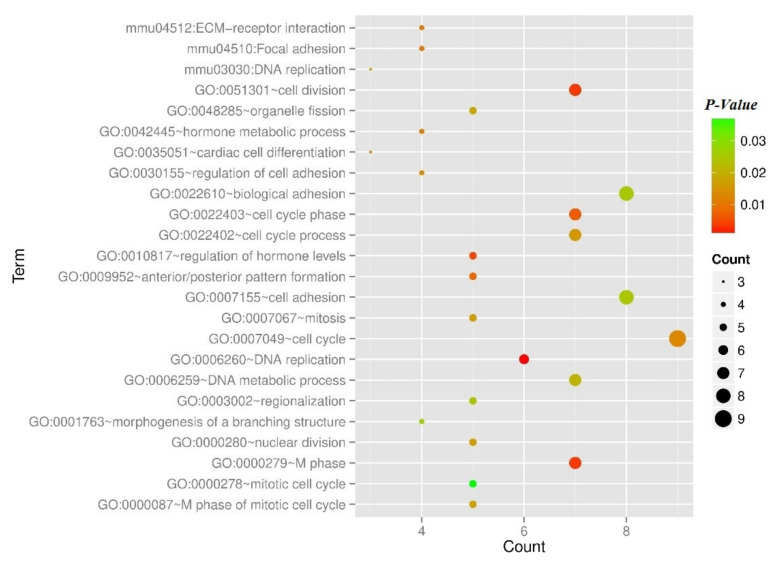
The scatterplot of the category of enriched GO terms and KEGG pathways for the DEGs in the co-expression network. Node size means gene count, color means *p*-value.

Moreover, GO and KEGG pathway enrichment analysis showed that a total of 21 significantly related GO BPs and 3 KEGG pathways were found for the DEGs in the co-expression network. DEGs in the co-expression network were significantly (*p*-value < 0.01) related to the cell cycle BPs and participated in ECM-receptor interaction (mmu04512), Focal adhesion (mmu04510), and DNA replication (mmu03030) pathways (Figure 6 and Table 3).

### 2.5. miRNA-DEGs-TF Regulatory Network Construction

**Figure 7:**
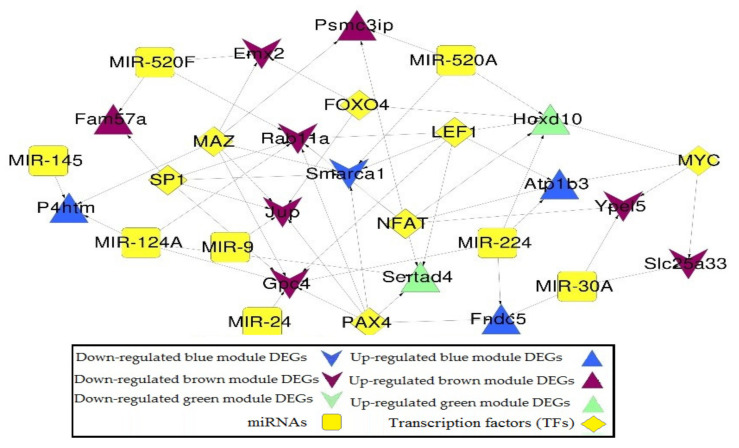
miRNA-DEG-TF regulatory network of DEGs in the co-expression network. Triangle and inverted triangles refer to up and down-regulated DEGs; blue, green, and brown nodes mean genes from the corresponding colored module. Yellow square and diamond mean miRNAs and TFs.

MicroRNAs (miRNAs) assume a pivotal role in controlling inborn and versatile immunity in humans and animals [22,23]. A total number of eight miRNAs and seven TFs that have the potential to regulate ODEGs in the co-expression network are listed in Table 4 and Table 5, respectively. Integration regulatory relationships were identified and constructed as a miRNA-DEGs-TF regulatory network between miRNA and DEGs, TFs and DEGs (Figure 7). In that network, 29 nodes (8 miRNAs, 7 TFs, and 14 DEGs), 7 down (1 blue, 6 brown) and 7 up-regulated (3 blue, 2 brown, and 2 green genes) and 56 edges (20 miRNA-DEGs regulation and 36 TFs-DEGs) have been confirmed (Table S4).

### 2.6. PCA for Genes in Regulatory Network

**Figure 8:**
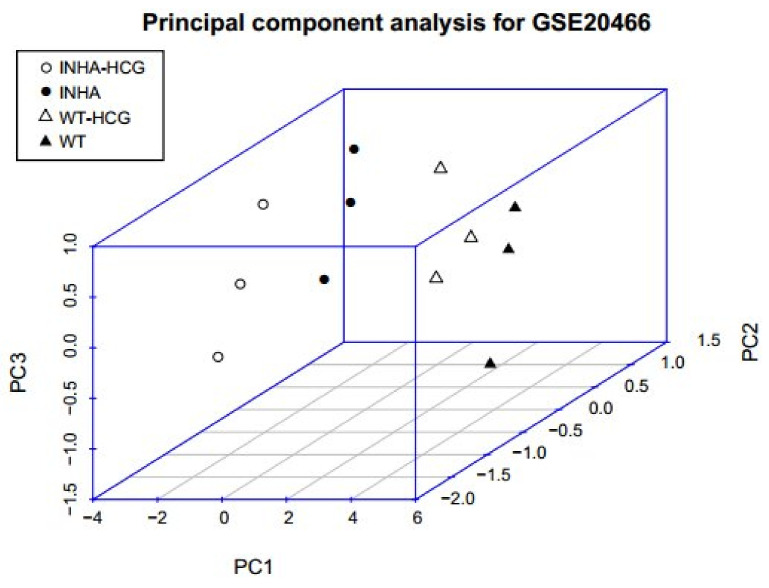
Three-dimensional distribution of samples based on PC1, PC2, and PC3. Black solid points and hollow points mean Inha−/− with and without hCG samples, and solid and hollow triangles mean WT with and without hCG samples.

To further refine the important genes, the PCA algorithm defined 14 genes in the regulatory network. The cumulative proportion of fitted top 3 principal components accounts for 94.64% of the total variance, which means that they can effectively describe the vast majority of input gene variables [20]. In general, the cumulative contribution rate of more than 80% is considered to have caught most of the input variable information. Figure 8 showed the differences in the top 3PCs between the two groups where significant differences (*p* < 0.01) were observed among the four different groups. Also, the gene contributions to the PCs were listed in Table 6, where there were 10 genes whose contribution rate (absolute value) was over 0.9.

## 3. Discussion

It is shown that Inhibin-α plays an important role in follicular development, oocyte development, cell differentiation, and finally reproduction. The Inhibin-α knockout/down female mouse can develop ovarian cancer and the LH and FSH may play a crucial role in GCs tumor development [24,25]. This study aimed to examine hCG-induced gene expression changes in different types of granulosa cells (WT and Inha−/− types). Also, it provided an important reference for the pathway mechanisms by showing that DEGs were different in WT/Inha−/− before and after hCG stimulation. The ODEGs were used as characteristic genes in hCG stimulation and Inhibin α knockout (Inha−/−) female mice granulosa cells. This observation is in agreement with FarmanUllah, Liang, Khan, Salim, Rehman, Khan, Talpur, Schreurs, Gouda, Khan and Shujun [22] who mentioned that ODEGs can effectively work as biomarkers for immune-related tumors. In which, 341 DEGs had high relevance, with a significant correlation coefficient (*p* < 0.000001) in both Inha−/− vs. WT group and Inha−/− (hCG) vs. WT(hCG) group which means that they could be used as characteristic genes in hCG stimulation and Inhibin α knockout (Inha−/−) female mice granulosa cells. Therefore, this study demonstrated for the first time that hCG induces the granulosa cells to excrete Inha through stimulating Fndc5, Sertad4, JUP, and Psmc3ip genes. In which, quantitative reverse transcriptase-polymerase chain reaction (qRT-PCR) on selected gene expression changes were observed in the gene array analysis verified the most important ODEGs of the knockout mice. Vasilache, et al. [26] mentioned that qRT-PCR microarray combined with modeling is an effective technique to detect the knockout mice’s important DEGs.

According to the analysis of KEGG pathway annotations, ODEGs were significantly enriched in eight biological pathways: DNA replication, Focal adhesion, and purine metabolism pathways which significantly enriched the GO term. In the biological process category, the genes were mainly enriched in GO terms associated with extracellular matrix and axon [27], for which, the R^2^ between the physiological phenotypes and each KEGG module for DEGs had a very high correlation coefficient (>0.8). Chen, et al. [28] mentioned that a positive regulator of the steroidogenesis pathway of FSH is essential for the granulosa cell proliferation, death, and differentiation in almost all cell types. The WGCNA algorithm was used to detect related modules and genes, significantly related to eight miRNAs; whereas, the seven TFs and regulatory networks were utilized to get regulated DEGs [29]. Finally, PCA differentiated the four groups under study to determine the importance of 14 regulated genes. Also, the top 3PCs between the two groups were significantly different (*p* < 0.01) among the four groups under study. Among them, the JUP gene was significantly related to cell adhesion (GO: 0007155), DNA metabolic process (GO:0006259), and biological adhesion (GO: 0022610), while Psmc3ip participated in the cell cycle (GO: 0022402) and M phase (GO: 0000279), and they all belonged to the brown module of the physiological phenotypes of WGCNA results, so they had close expression relationship. Chen, et al. [30] reported that the JUP form is a member of the catenin family that can affect various processes such as proliferation, migration, and differentiation by mediating cellular adhesion. Thus, the mutation in its gene is associated with several gene-related diseases. [30]. In addition, Psmc3ip (also known as GT198) is used as a unique tumor marker suppressor gene for the mutant cells in ovarian cancer. Psmc3ip protein has been shown as a steroid hormone receptor regulator and also as a crucial factor in DNA repair [31]. Thus, studying such genes could facilitate the complex mission of dealing with ovarian cancer.

Additionally, the result suggested that Inhibin α knockout and hCG stimulation can down-regulate the expression of JUP and up-regulate Psmc3ip. In which, the co-expression Network had 153 DEGs expression correlation coefficient from WGCNA algorithm with R^2^ > 0.8 with 35 down-regulated genes and 87 up-regulated genes. Moreover, JUP forms distinct complexes with cadherins and desmosomal cadherins through an amino acid motif called the armadillo repeat, which can affect the diverse processes and modulate the function of extracellular ligands [32,33]. It also showed that JUP and Psmc3ip genes had close relationships in both expression patterns and functions in the Inha−/− hCG stimulation female mice granulosa cells. Similarly, the activity of the Psmc3ip gene is revealed to have a crucial role in ovarian dysgenesis and male fertility in mammalians [34,35]. Thus, the data analysis detected DEGs and relevant biological functions after the knockdown of the Inha and associated gene expression for further research guidance in mammalian reproduction.

## 4. Materials and Methods

### 4.1. Experimental Animals

A number of 100 specific-pathogen-free (SPF) mice were grouped (25 mice WT, 25 mice Inha−/−, 25 mice WT (hCG), and 25 mice Inha−/−(hCG)) according to Hofland, et al. [36]. In which, 21 to 23-day-old WT and Inha−/− female mice were injected with 5 IU hCG for 6 h to stimulate hCG groups and granulosa cells with and without hCG stimulation which were collected from 2 genotypes (WT and Inha−/−) according to National Institutes of Health (NIH) Guidelines for the Care and use of Laboratory Animals, USA (Approval ID: SCXK Hubei 20080005).

### 4.2. Data and Experimental Design

**Figure 9:**
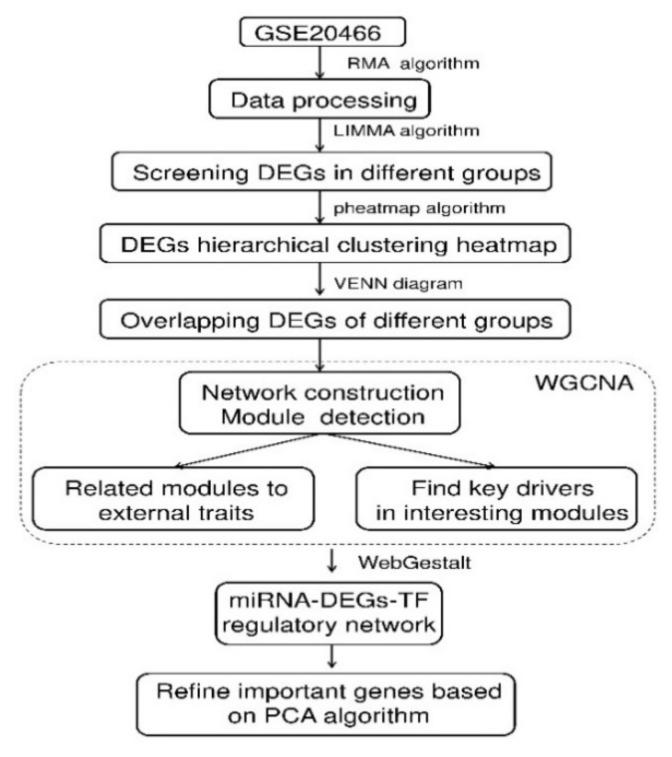
Schematic illustration of the analysis strategy.

A schematic diagram of the overall research procedure for data analysis is shown in Figure 9. Dataset and description.

The target gene expression profiles were downloaded from NCBI Gene Expression Omnibus (GEO; http://www.ncbi.nlm.nih.gov/geo/ accessed on 1 February 2011) through accession number GSE20466 (Platform: GPL1261 [Mouse430_2] Affymetrix Mouse Genome 430 2.0 Array), which contained 12 samples in total [37,38].

### 4.3. Data Reprocessing and Differentially Expressed Genes (DEGs) Screening

**Figure 10:**
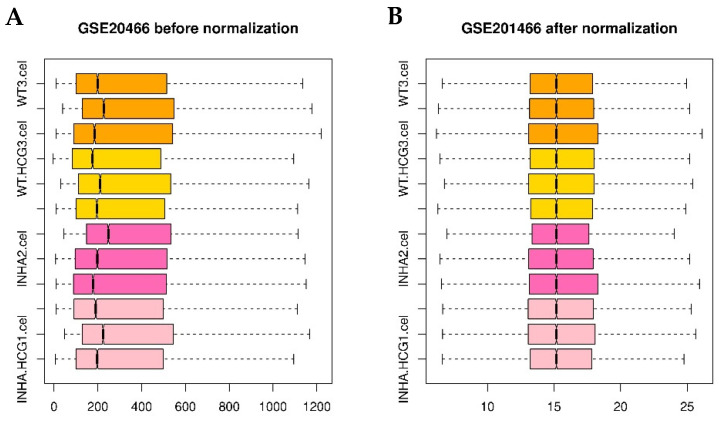
Boxplot of GSE20466 data preprocessing before (**A**) and after normalization (**B**). Pink, hot pink, gold, and orange boxes refer to Inha−/−(hCG), Inha−/−, WT(hCG), and WT samples.

**Figure 11:**
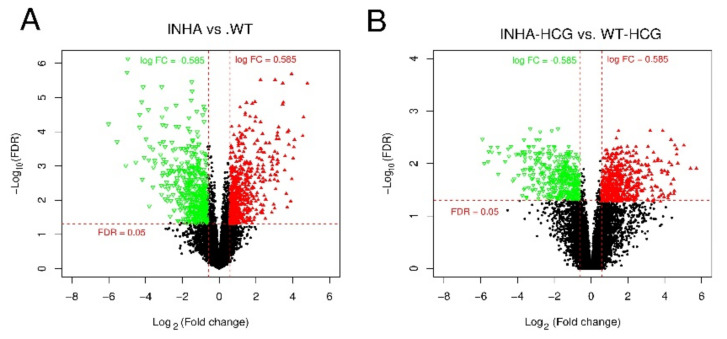
Volcano plot of DEGs in group Inha−/− vs. WT (**A**) and Inha−/− (hCG) vs. WT (hCG) (**B**). The red triangle and green inverted triangle refer to up and down-regulated DEGs; the Red horizontal dot line means FDR = 0.05 cutoff line, and two red vertical dot lines mean logFC = 1 and logFC = −1 cutoff line.

The main objective of this part was to initially normalize the datasets’ differences and functions. The data before and normalization were shown in Figure 10, and the detailed normalized gene expression data can be found in Table S5. Only those genes meeting FDR < 0.05 and |log2 FC (fold change)| > 1 were chosen as DEGs from each group. In Inha−/− vs. WT and Inha−/− with hCG vs. WT groups only 1074 and 931 DEGs were identified based on the cut-off criteria and showed in volcano plots for WT (Figure 11A), and Inha−/− (Figure 11B) respectively. The list of DEGs could be found in Table S6 (Supplementary Materials).

Raw CEL files and annotation files were downloaded, and the gene expression data of all samples were preprocessed via background correction, quantile normalization, and probe summarization using the Robust Multi-array Average (RMA) algorithm (http://www.bioconductor.org/packages/release/bioc/html/affy.html, accessed on 1 February 2022) in R 3.4.1 (R Studio, USA). Linear Models of Microarray Data package (LIMMA, version 3.32.5) from the link http://www.bioconductor.org/packages/release/bioc/html/limma.html (accessed on 1 February 2022) was used to identify DEGs [39].

### 4.4. Hierarchical Clustering and Comparison Analysis of Selected DEGs in Different Groups

The expression of selected DEGs in Inha−/− vs. WT and Inha−/− (hCG) vs. WT (hCG), Inha−/− vs. Inha−/− (hCG), and WT vs. WT (hCG) were used to generate a hierarchical clustering image by heatmap (version 1.0.8) package in R 3.4.1 (RStudio; http://www.cran.r-project.org/web/packages/pheatmap/, accessed on 1 February 2022) [40,41]. Then, the identified DEGs were compared in the two groups and the ODEGs were selected by using VennDiagram package R 3.4.1 (http://www.cran.r-project.org/web/packages/VennDiagram/, accessed on 1 February 2022). After that, Pearson Correlation Coefficient (PCC) was used for further studying the ODEGs correlations following Huang da, et al. [42].

### 4.5. Enrichment Analysis for the Overlapping DEGs

To explore the functions of ODEGs and their pathways, the DAVID version 6.8 (Database for Annotation, Visualization and Integrated Discovery; http://www.david.ncifcrf.gov/, accessed on 1 February 2022) database was used to perform GO (Go Ontology) and KEGG (Kyoto Encyclopedia of Genes and Genomes) pathway enrichment analyses for ODEGs. The *p*-value < 0.05 and gene count ≥ 2 were set as the cut-off criteria. Furthermore, the category of enriched GO, KEGG terms, and the gene number were displayed as scatterplots by the ggplot2 package in R3.4.1 (http://www.cran.r-project.org/web/packages/ggplot2/, accessed on 1 February 2022).

### 4.6. Physiological Phenotypes-Related Modules and Genes Identification Based on WGCNA

Weighted Gene Co-expression Network Analysis (WGCNA) algorithm was used to investigate the co-expression modules and genes which were related to phenotypes through the WGCNA package (version 1.61) (http://www.cran.r-project.org/web/packages/WGCNA/index.html, accessed on 1 February 2022).

### 4.7. Co-Expression Network Construction

Based on the results of the WGCNA algorithm, only gene pairs with expression correlation coefficient > 0.8 were used to construct a gene co-expression network which was then built by Cytoscape3.2.0 (http://www.cytoscape.org/, accessed on 1 February 2022). Also, GO and KEGG pathway enrichment analysis for the genes in the co-expression network was made.

### 4.8. miRNA-DEGs-TF Target Regulatory Network Analysis

WEB-based Gene Set Analysis Toolkit (WebGestalt; http://www.webgestalt.org/option.php, accessed on 1 February 2022) was used to search Transcription Factors (TFs) and miRNAs that regulated the DEGs in co-expression networks. Besides, *p*-value < 0.05 was set as the significance cut-off criteria. As a result of TFs and miRNAs were integrated and then identified in the miRNA-DEGs-TF regulatory network. The regulatory network consisting of DEGs, miRNAs, and TFs was then constructed and visualized by Cytoscape3.2.0 (http://www.cytoscape.org/, accessed on 1 February 2022).

### 4.9. Principal Component Analysis (PCA) for Genes in the Regulatory Network

In order to refine genes and get the most specific ones, we further narrowed the gene range by using the PCA algorithm in the psych package (version 1.7.5) in R3.1.4 (http://www.cran.r-project.org/web/packages/psych/, accessed on 1 February 2022). Then scatterplot3d package (version 0.3-40) (http://www.cran.r-project.org/web/packages/scatterplot3d/, accessed on 1 February 2022) was used to display the effect of PCA based on the top 3 components: PC1, PC2, and PC3.

## 5. Conclusions

In this study, 1074 and 931 DEGs aggregates were identified in inha and wild-type. Through bioinformatics investigation 8 miRNAs, 7 TFs and 14 DEGs and 7 up-regulated genes with 20 miRNA-DEGs regulation and 36 TFs-DEGs were confirmed. This study provides potential key information for using ODEGs as biomarkers for granulosa cell cancer regulation. Also, further integration of the DEGs and the TF related to the immune response can facilitate the development of the target drugs for controlling the transcription pathways of the inhibin-deficient females.

## Figures and Tables

**Table 1:** Enriched GOs and KEGG pathways for overlapped genes.

Category	Term	Count	*p*-Value
Biology Process	GO:0022402~cell cycle process	17	4.05 × 10^−6^
	GO:0006259~DNA metabolic process	17	8.56 × 10^−4^
	GO:0022403~cell cycle phase	16	1.79 × 10^−4^
	GO:0000279~M phase	15	1.31 × 10^−4^
	GO:0010033~response to organic substance	15	2.53 × 10^−2^
	GO:0006260~DNA replication	13	4.14 × 10^−6^
	GO:0051301~cell division	11	1.21 × 10^−2^
	GO:0001568~blood vessel development	10	1.37 × 10^−2^
	GO:0001944~vasculature development	10	1.58 × 10^−2^
	GO:0001501~skeletal system development	10	3.33 × 10^−2^
Cellular Component	GO:0005578~proteinaceous extracellular matrix	19	5.11 × 10^−6^
	GO:0005615~extracellular space	19	4.03 × 10^−3^
	GO:0044427~chromosomal part	15	1.49 × 10^−3^
	GO:0005694~chromosome	15	7.06 × 10^−3^
	GO:0044454~nuclear chromosome part	8	2.25 × 10^−3^
	GO:0000228~nuclear chromosome	8	5.51 × 10^−3^
	GO:0005657~replication fork	6	5.21 × 10^−5^
	GO:0000793~condensed chromosome	6	4.05 × 10^−2^
	GO:0042383~sarcolemma	5	9.21 × 10^−3^
	GO:0030018~Z disc	4	4.69 × 10^−2^
Molecular Function	GO:0019838~growth factor binding	6	4.09 × 10^−3^
	GO:0008094~DNA-dependent ATPase activity	4	1.80 × 10^−2^
	GO:0016875~ligase activity, forming carbon-oxygen bonds	4	3.32 × 10^−2^
	GO:0004812~aminoacyl-tRNA ligase activity	4	3.32 × 10^−2^
	GO:0005520~insulin-like growth factor binding	3	4.08 × 10^−2^
KEGG Pathway	mmu03030:DNA replication	8	1.35 × 10^−6^
	mmu03430:Mismatch repair	5	4.27 × 10^−4^
	mmu04512:ECM-receptor interaction	8	4.34 × 10^−4^
	mmu03420:Nucleotide excision repair	6	6.87 × 10^−4^
	mmu03440:Homologous recombination	4	9.92 × 10^−3^
	mmu04510:Focal adhesion	9	1.73 × 10^−3^
	mmu03410:Base excision repair	4	2.87 × 10^−2^
	mmu00230:Purine metabolism	7	4.71 × 10^−2^

**Table 2:** Correlation between physiological phenotypes and each module genes.

Color	Gene Count	Correlation Coefficient (R^2^)
blue	65	0.9218561
green	28	0.9203381
brown	60	0.8912573
turquoise	81	0.8894315
yellow	51	0.889348
grey	58	0.8815949

**Table 3:** Enriched GOs and KEGG pathways for co-expression network genes.

Parameter	Term	Count	*p*-Value
Biology Process	GO:0007049~cell cycle	9	0.012955
	GO:0007155~cell adhesion	8	0.02503
	GO:0022610~biological adhesion	8	0.025243
	GO:0051301~cell division	7	0.003211
	GO:0000279~M phase	7	0.003325
	GO:0022403~cell cycle phase	7	0.00678
	GO:0022402~cell cycle process	7	0.015593
	GO:0006259~DNA metabolic process	7	0.021132
	GO:0006260~DNA replication	6	0.001106
	GO:0010817~regulation of hormone levels	5	0.004264
	GO:0009952~anterior/posterior pattern formation	5	0.007972
	GO:0000280~nuclear division	5	0.016588
	GO:0007067~mitosis	5	0.016588
	GO:0000087~M phase of mitotic cell cycle	5	0.017769
	GO:0048285~organelle fission	5	0.018688
	GO:0003002~regionalization	5	0.024455
	GO:0000278~mitotic cell cycle	5	0.03702
	GO:0042445~hormone metabolic process	4	0.011174
	GO:0030155~regulation of cell adhesion	4	0.012565
	GO:0001763~morphogenesis of a branching structure	4	0.026619
	GO:0035051~cardiac cell differentiation	3	0.012461
KEGG pathway	mmu04512:ECM-receptor interaction	4	0.010701
	mmu04510:Focal adhesion	4	0.009668
	mmu03030:DNA replication	3	0.015987

**Table 4:** Related miRNAs list.

miRNA	ID	*p*-Value	FDR
mmu_TGCCTTA,MIR-124A	DB_ID:590	9.65 × 10^−3^	0.0014
mmu_GTGACTT,MIR-224	DB_ID:524	0.0002	0.0014
mmu_CTCTGGA,MIR-520A	DB_ID:484	0.0036	0.0126
mmu_ACCAAAG,MIR-9	DB_ID:588	0.0029	0.0126
mmu_ACTGAAA,MIR-30A	DB_ID:464	0.0065	0.0182
mmu_CTGAGCC,MIR-24	DB_ID:539	0.0107	0.0194
mmu_AACTGGA,MIR-145	DB_ID:614	0.0101	0.0194
mmu_AAGCACT,MIR-520F	DB_ID:615	0.0111	0.0194

**Table 5:** Related TFs list.

TF	ID	*p*-Value	FDR
PAX4	DB_ID:1830	8.59 × 10^−6^	2.58 × 10^−5^
MAZ	DB_ID:1815	7.72 × 10^−6^	2.58 × 10^−5^
MYC	DB_ID:1819	4.99 × 10^−6^	2.58 × 10^−5^
NFAT	DB_ID:1822	1.40 × 10^−5^	3.15 × 10^−5^
FOXO4	DB_ID:1801	3.72 × 10^−5^	6.70 × 10^−5^
SP1	DB_ID:1837	2.00 × 10^−4^	3.00 × 10^−4^
LEF1	DB_ID:1813	4.00 × 10^−4^	4.00 × 10^−4^

**Table 6:** Gene contributions to PC1-3.

Gene	Contribution to PC1-3
Fndc5	0.97
Sertad4	0.97
Atp1b3	0.96
Fam57a	0.95
P4htm	0.89
Hoxd10	0.85
Psmc3ip	0.85
Rab11a	−0.85
Ypel5	−0.9
Emx2	−0.91
Jup	−0.92
Gpc4	−0.93
Slc25a33	−0.94
Smarca1	−0.94

## Data Availability

Available upon request.

## Supplementary Materials

The following supporting information can be downloaded at: https://www.mdpi.com/article/10.3390/molecules27175595/s1.
